# Comparison of compensatory strategies and gait deviations in unclassified and type 1 unilateral cerebral palsy

**DOI:** 10.1038/s41598-026-40523-5

**Published:** 2026-02-20

**Authors:** Stefanos Tsitlakidis, Nicholas A. Beckmann, Johannes Weishorn, Sebastian I. Wolf, Pit Hetto, Paul Mick

**Affiliations:** Department of Orthopaedics, Heidelberg University Hospital, Schlierbacher Landstrasse 200a, 69118 Heidelberg, Germany

**Keywords:** 3D-instrumented gait analysis, unilateral cerebral palsy, compensatory strategies, transversal asymmetry, Winters classification, sound/uninvolved limb, Outcomes research, Paediatric research

## Abstract

Different classification systems were described in the past for unilateral Cerebral Palsy (CP) focusing on gait deviations of the involved limbs mainly in the sagittal plane. However, there is a high percentage of patients that remain unclassified. Particularly in unilateral CP due to the naturally given asymmetry, the gait deviation causes compensatory movement patterns, thus affecting the sound limb. The objective of this work was a comprehensive assessment of gait deviations (involved and sound limb) in patients with unilateral CP, particularly in comparison between unclassified patients, type 1 hemiplegia and typically developing individuals. 47 individuals with unilateral CP (15 unclassified, 32 type 1) were included. Differences between those two groups and particularly compared to typically developing individuals, including pelvis and trunk and all planes/degrees of freedom, were analyzed using instrumented 3D gait analysis (IGA). Most remarkably, gait deviations due to anatomical and functional leg length discrepancies (LLD) and pelvic rotation were found in all participants, still not considered for classification of patients with unilateral CP. Alterations due to LLD and transversal asymmetry are additional predominant and highly significant modulations with respect to compensatory movement strategies in all patients with unilateral CP and should be considered for classification and treatment recommendations.

## Introduction

Cerebral palsy (CP) is a complex and most common neuromuscular condition in childhood that leads to a variety of pathological gait patterns and compensatory mechanisms^1–4^. The extent of the secondary movement disorder (secondary deviation of the involved limb) is dependent on the primary brain lesion^1,5–8^. Particularly in unilateral CP, the secondary deviation itself causes compensatory movement patterns or at least adaption (tertiary deviation) of the sound limb^9–12^. The naturally given asymmetry and consecutive highly complex movement patterns in unilateral CP on the one hand, and the ability of the sound limb to compensate on the other hand, affect the overall gait function^11–14^. Furthermore, overall propulsion and stability of walking is dependent on the functional capabilities of the sound limb^15,16^.

However, just a few reports characterizing gait patterns in patients with unilateral CP are available in the literature^17–20^. Different classification systems have been described in the past; the Winters, Gage, and Hicks (WGH) being the most commonly used classification system focusing on morphologic aspects of the gait pattern^18,20–22^. It includes four subtypes taking only sagittal plane kinematics of the whole lower limb (especially ankle joint, knee and hip joint partially, disregarding pelvic and trunk movements) into account^22^. Still, there is a high percentage of unclassified patients using the WGH classification system^18,20,23–25^. As a consequence, it was concluded that those individuals were less or even negligible involved and the closest to physiological gait patterns, yet not normal^18,20,23^, though showing deviations in other planes than commonly considered^25^. Coronal and transversal plane deviations as well as compensatory adaptions/strategies of the sound limb in unilaterally affected individuals are mainly not considered in established classification systems^10,21^. Nonetheless, transversal plane malalignment (particularly internal hip rotation of the involved limb) and compensatory deviations (pelvic protraction on the uninvolved side) are of importance for sagittal alignment (foot orientation), as these factors influence joint/muscle leverage (e.g. hip abductor weakness due to malrotation) and thus for a sufficient propulsion of walking (e.g. altered plantarflexion moment due to malrotation)^24,26–31^. Particularly, possible tertiary (compensatory) deviations of the uninvolved limb in unilateral CP have received little attention so far, though, it is clear that the gait deviations of the involved limb influence the gait pattern of the sound limb^10,11,32^. Insights into tertiary gait deviations of the sound limb might lead to a better and holistic understanding of the pathophysiology of gait deviations in unilateral CP and may have an impact on treatment approaches, as treatment planning in unilateral CP, so far, mainly considers and is conducted on the involved side.

Therefore, our objective was a detailed assessment of tertiary gait deviations of the sound limb (kinematic features including all degrees of freedom) particularly between patients classified as WGH type 1, unclassified patients (so called “0-group”) and typically developing individuals (TD). Special attention was paid to differences between the unclassified and patients with WGH type 1, in order to assess for further and possibly characteristic subgroup-specific deviations.

## Patients & methods

The current work was conducted as a database study including patients exclusively with unilateral CP after approval by the local ethics committee (S-198/2019). The study was conducted in accordance with the current version of the Helsinki declaration. Written and oral consent was given by all study participants and/or their legal guardians.

Our *inclusion criteria* were: patients exclusively with unilateral spastic CP, classified as WGH type 1 (primarily drop foot in the swing phase with consecutive equinus deformity at initial contact) or unclassified by the WGH system (missing drop foot deformity, gait pattern yet not normal), no previous surgery of the lower limbs, no Botulinumtoxin–A injections within the last six months, no additional condition affecting the motor ability and performance of the child.

### Study population

47 individuals with unilateral CP (22 female, 25 male) with a mean age of 16.1 ± 8.9 years at the time of instrumented 3D gait analysis (IGA) were included. More specific demographic and anthropometric characteristics are given in Table 1. The reference data were derived from a group of typically developing individuals (TD) from our gait laboratory database. The TD reference group consisted of 26 participants (52 limbs) with a mean age of 15.1 ± 5.9 years.

**Table 1:** Demographic and anthropometric characteristics including the corresponding statistics.

	*n*	age [years]	ratio f: m	leg length involved mean ± SD [cm]	leg length uninvolved mean ± SD [cm]	leg length discrepancy mean ± SD [cm]
WGH unclass.	15	16.1 ± 7.4	5:10	78.7 ± 9.4	79.9 ± 9.4	1.2 ± 0.9
WGH type 1	32	16.1 *±* 9.5	17:15	80.3 ± 8.8	81.2 ± 9.0	0.9 ± 0.9
*total*	*47*	*16.1 ± 8.9*	*22:25*	*79.8 ± 9.0*	*80.8 ± 9.1*	*1.0 ± 0.9*
*TD*	*26*	*15.1 ± 5.9*	*13:13*	*-*	*82.3 ± 8.3*	*0.5 ± 0.4*
*p-values* *(type1 vs. unclass.)*		0.984		*0.588*	*0.652*	*0.407*

### Gait analysis

IGA was performed from 2006 to 2017 using a 120-Hz 9-camera system (Vicon, Oxford Metrics, Oxford, UK) and two piezoelectric force plates (Kistler, Winterthur, Switzerland) read out by a sampling frequency of 1080 Hz (9 times the camera frequency) were used. Reflective markers were applied to bony landmarks according to the Plug-In Gait lower body model and protocol^33,34^. In this procedure, the knee axis was determined by the examiner via a knee alignment device. Four additional markers on the subjects’ shoulder girdle (processus spinosus of the 7th cervical vertebra, left and right acromion, and incisura jugularis) were used to observe trunk motion in relation to the global reference frame^35^. The participants walked a seven-meter walkway barefoot and at a self-selected speed. Data on leg length discrepancies (LLD) were collected routinely as part of our standard gait laboratory protocol for the clinical examination of all participants prior to every gait analysis. Leg lengths were measured from the superior anterior iliac spine to the medial malleolus using a flexible tape measure.

### Data analysis

Kinematic parameters were processed via commercial software by Vicon using the Plug-In Gait model averaging at least five strides^34,36^. For visual inspection of stride-to-stride consistency as well as time normalization of gait data to the gait cycle (GC in %), lab-specific software codes were used on the basis of Matlab R2018b (MathWorks, Natick, MA, USA). The following kinematic features considering all lower limb joints as well as the pelvis and trunk and all degrees of freedom have been included for further analysis:

- Trunk tilt, trunk obliquity and trunk rotation.
- Pelvic tilt, pelvic obliquity and pelvic rotation;
- Hip flexion/extension, hip abduction/adduction and hip rotation;
- Knee flexion/extension, knee valgus/varus and knee rotation;
- Ankle flexion/extension, ankle valgus/varus and foot progression.

Foot progression, describing the orientation of the foot’s long axis in relation to the gait direction, has been chosen instead of ankle rotation, since this parameter is given more clinical importance.

The collected parameters were compared against each other in order to investigate for potential differences between the subgroups, in order to assess for specific characteristics and deviations from the gait of typically developing (TD) individuals.

According to Perry et al., a gait cycle was divided into the following sub-phases: loading response (LR), mid stance (MSt), terminal stance (TSt), preswing (PSw), initial swing (ISw), mid swing (MSw) and terminal swing (TSw)^37^.

### Statistical analysis

Data were analyzed using Microsoft Excel (Microsoft, Redmond, WA, USA) and Matlab R2018b (MathWorks, Natick, MA, USA). For descriptive statistics the mean and the standard deviation (SD) were calculated and displayed graphically. Comparative statistics of subgroup-specific demographic and anthropometric characteristics included two-tailed student´s t-test. For comparative continuous statistics, one-dimensional statistical parametric mapping (SPM) was performed with ANOVA-1D followed by Bonferroni’s post hoc-test throughout the whole gait cycle using custom scripts in Matlab based on previous own work and on the work of Pataky et al.^38–41^ The level of significance was set at *p* < 0.05.

## Results

Further demographic and anthropometric characteristics of the study population including the results of the statistical analysis are given in Table 1. There were no statistically significant differences between the unclassified patients and patients with a WGH type 1. Most importantly, there was no significant difference in age, so group equality was ensured in this regard (Table 1).

**Fig. 1:**
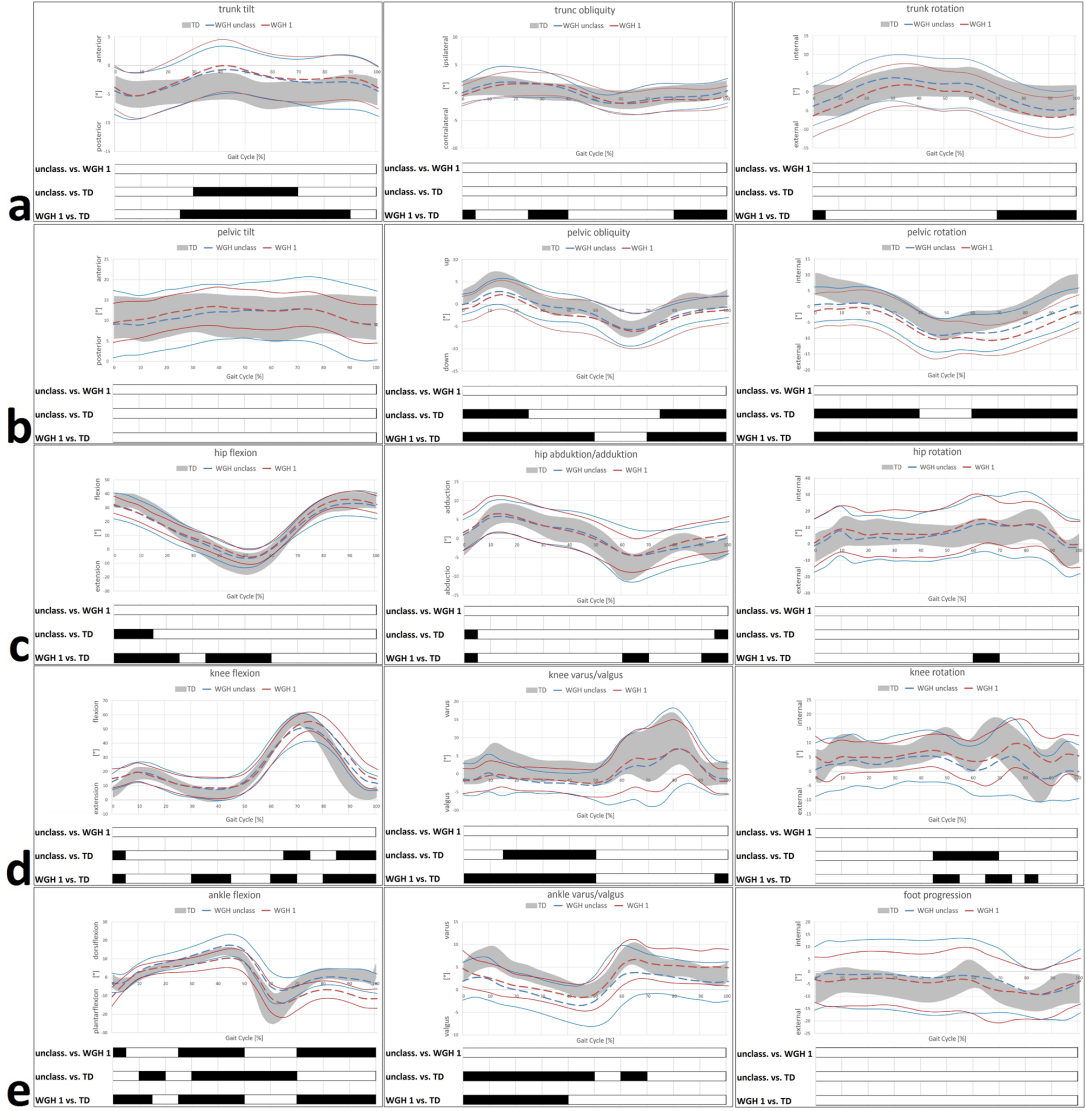
Subtype-specific kinematics of the involved limb. Trunk kinematics (**a**); pelvic kinematics (**b**); hip kinematics (**c**); knee kinematics (**d**); ankle/foot kinematics (**e**). **TD** group (age-matched typically developing individuals) of the gait laboratory data-base. Black bars represent the results of **SPM** and indicate significant differences during the corresponding parts (in %) of the gait cycle.

**Fig. 2:**
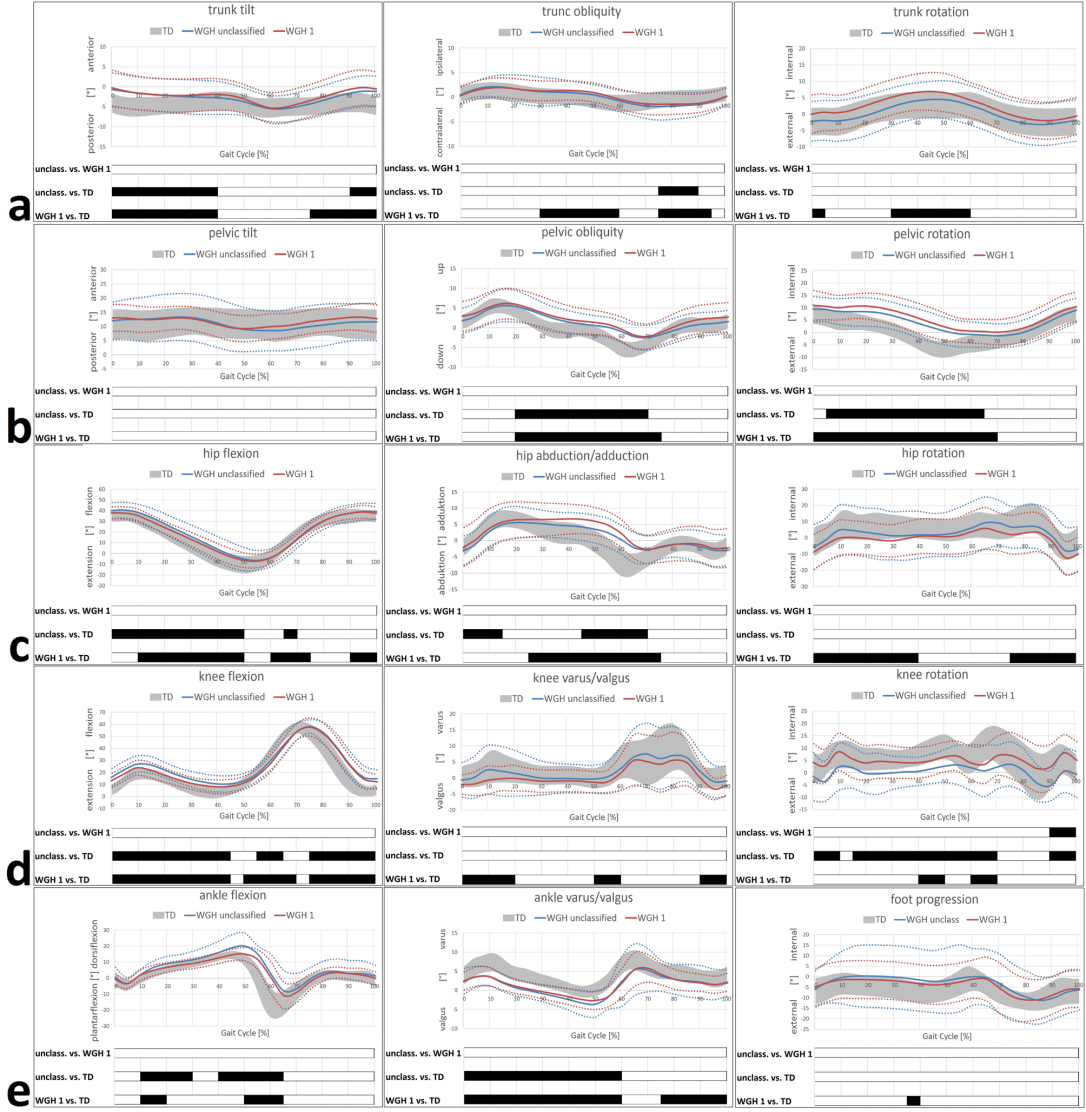
Subtype-specific kinematics of the sound limb. Trunk kinematics (**a**); pelvic kinematics (**b**); hip kinematics (**c**); knee kinematics (**d**); ankle/foot kinematics (**e**). **TD** group (age-matched typically developing individuals) of the gait laboratory data-base. Black bars represent the results of **SPM** and indicate significant differences during the corresponding parts (in %) of the gait cycle.

There were no statistically significant differences between the unclassified and WGH type 1 patients in trunk kinematics, while both groups differ in this regard from the TD (Figs. 1 and 2). Most remarkable deviations concerning trunk kinematics was an increased anterior tilt, in both the unclassified and WGH type 1 patients, from terminal TSt to ISw on the involved side (corresponding to an increased anterior tilt from TSt to MSt on the sound side). Additionally, pronounced trunk obliquity and rotation towards the involved side during TSt/PSw and parts of MSw was evident.

At pelvic level, pelvic obliquity and rotation towards the involved side was evident in the unclassified and WGH type 1 patients during the majority of the gait cycle. Again, there were no statistically significant differences between the unclassified and WGH type 1 patients (Figs. 1 and 2).

Considering hip kinematics in all three planes, no differences between the unclassified and WGH type 1 patients were found (Figs. 1 and 2). Pronounced hip flexion mainly during the stance phase (particularly WGH type 1) was evident on the involved (secondary deviation), but as well on the uninvolved side. Furthermore, pronounced hip adduction particularly from TSt to ISw on the sound side was found, which is concomitant and as a result to pelvic obliquity. Altered hip rotation was noticeable mainly in WGH type 1 on the sound side during stance phase and MSw/TSw.

Knee kinematics as well did not show any differences between the unclassified and the WGH type 1 patients (Figs. 1 and 2). Increased knee flexion was evident for both groups on the involved and sound side. Knee valgus particularly during stance phase was found on the involved side. However, particularly the unclassified patients showed external knee rotation on the sound side.

At ankle level, most remarkable deviations and especially differences between the unclassified and the WGH type 1 patients were found in the sagittal plane (Figs. 1 and 2). Here, the unclassified patients showed increased dorsiflexion during the majority of the gait cycle on the involved side, whereas WGH type 1 patients showed increased plantarflexion. Additionally, WGH type 1 patients showed drop foot, which is missing in the unclassified patients. Moreover, increased dorsiflexion was seen in both groups on the uninvolved side. Beyond that, ankle valgus during stance phase and physiological foot progression were seen in both groups on the involved and sound side.

## Discussion

Little is known about the effect of secondary gait deviations of the involved limb on the gait pattern/compensatory movements (tertiary deviation) of the sound limb in individuals with unilateral CP due to the naturally given asymmetry, particularly in the unclassified patients.

The objective of this work, on the one hand, was the assessment of secondary and especially tertiary gait deviations in patients with unilateral CP including both, the involved and sound side. On the other hand, special attention was given to differences particularly between the unclassified patients and WGH type 1 as well as compared to the TD including all three planes and all degrees of freedom as well as trunk and pelvic kinematics.

With regard to secondary/tertiary gait deviations, most remarkably, our results suggest that, in the sagittal plane increased hip and knee flexion mainly in WGH type 1 patients on the involved side was evident as a secondary and tertiary deviation due to equinus at initial contact. Predominantly, drop foot in the WGH type 1 patients was evident causing knee flexion on the involved side, in order to ensure ground clearance. Neither vaulting, nor pelvic hike were found to be apparent as a compensation for drop foot in WGH type 1. Interestingly, the unclassified patients showed increased ankle dorsiflexion throughout the majority of the gait cycle, which together with increased knee flexion suggests a crouch gait pattern. Increased hip, knee and ankle (dorsi-)flexion were evident on the sound side in order to compensate for anatomical and functional leg length discrepancy (LLD) and to reduce bilateral asymmetry. Here, our results suggest that knee flexion might be the most important factor for sufficient ground clearance, as it was the most pronounced and predominant for the longest period of the gait cycle. In the coronal plane, trunk/pelvic obliquity towards the involved side can be considered as a consequence of (anatomical and functional) LLD. Moreover, trunk obliquity (trunk lean) is considered to be a sufficient but energy-consuming compensatory mechanism to unload weak hip abductors^17,35,42,43^. In the transversal plane, pelvic rotation towards the involved side can be considered as compensatory to maintain neutral foot progression on the involved side. As a consequence, external hip rotation was noticeable mainly in WGH type 1 on the sound side, whereas the unclassified patients showed external knee rotation on the sound side, in order to compensate for pelvic rotation and to restore neutral foot progression on the sound side.

Our results are supported by the findings of other authors^11,12,23,24,42,44^. Increased hip and knee flexion, increased dorsiflexion during swing and stance and valgus foot deformity of the uninvolved limb were reported to be compensatory for LLD in order to reduce the functional leg length of the sound limb and thus to improve symmetry at pelvic level^10,32,45^. There are numerous kinematic-based studies that demonstrate a direct correlation between LLD and gait deviations. LLD leads to clinically relevant gait asymmetries, in particular changes in pelvic position (pelvic obliquity) and functional shortening of the longer limb. These adaptations are comparable to those seen in TD, but in CP they are often more pronounced and occur in combination with other pathological gait patterns of the involved limb^46–48^. Furthermore, transversal plane deviations have been described, yet not considered sufficiently^21,23^. Our findings suggest that, a transversal asymmetry (particularly pelvic rotation) even seen in the unclassified patients is one of the predominant gait deviations in unilateral CP. Pelvic rotation was deemed as a compensation mechanism to restore foot progression on the involved side^23^(underlining the importance of the transversal plane for gait function and walking capability), while the sound limb is able to compensate excessive pelvic asymmetry and to maintain neutral foot progression on the sound side, thus reducing bilateral asymmetry^11,12,23^. However, limited compensations on the sound side if the anatomic alignment was significantly asymmetric were described and concluded as a reason why transversal plane changes of the pelvis after femoral rotation osteotomy are unpredictable^12^.

Regarding the high number of unclassified patients, our results suggest that significant and relevant differences between the unclassified and WGH type 1 patients only concern sagittal plane ankle kinematics on the involved side. Here, the unclassified showed no equinus/drop foot, but increased ankle dorsiflexion compared to the TD, which can be assumed as the main reason why these patients are not classifiable using the WGH classification system. Moreover, compared to the TD the unclassified showed pelvic obliquity/rotation towards the involved side, hip, knee and ankle (dorsi-)flexion and increased knee external rotation and on the sound side. Even using the evolved classification by Rodda et al.^49^, which partly considers pelvic rotation (just for type 4 hemiplegia) these patients still remain unclassified, again emphasizing the relevance and significance particularly of transversal asymmetry in all patients with unilateral CP.

This current work represents the only holistic assessment (all levels, all planes of freedom) of gait disorders in unilateral CP considering the involved and sound limb, as a basis for further studies particularly focusing on transversal plane malalignment, thus allowing for a better understanding, evolved classification systems and improving treatment recommendations. From a clinician´s point of view, focusing on pelvic asymmetry and taking this into consideration, even in mildly involved patients with unilateral CP (unclassified and WGH type 1), improvement of gait function could be achieved by addressing the transversal malalignment. E.g. femoral derotation osteotomy (FDO) has been shown to improve frontal hip moments and restore pelvic symmetry^30,50–53^.

The main limitations of this current work is the absence of a differentiated multisegment foot model for further and more precise assessment of foot deformities. Concerning pelvic asymmetry and transversal plane deviations in general, soft tissue artefacts and marker displacement are possible and critical factors affecting the accuracy of the method, which represents a technical difficulty that, however, cannot be addressed using another method/model or marker protocol. Furthermore, methodological differences among laboratories could lead to different gait measures compromising inter-study comparability.

## Conclusion

LLD and transversal asymmetry (particularly pelvic rotation) are additional predominant and highly significant modulations in all patients with unilateral CP, even in the mildly involved. Restoring physiological gait in mildly involved unclassified individuals could be achieved by correction of the transversal asymmetry and LLD. More attention and consideration, particularly in terms of an individualized therapy decision-making, should be given taking the tertiary gait deviations of the sound limb into account, as they predispose the sound limb for or at least might be associated with secondary conditions as osteoarthritis in this vulnerable population.

As a consequence, therefore, pelvic rotation should be assessed in all patients with unilateral CP and, in terms of a modification and extension of the WGH or Rodda classification, should also be considered even in mildly affected (unclassified) patients as part of the decision-making process.

In this regard, future studies should strive for subtype specific transversal malalignment assessment (static vs. dynamic, femoral vs. tibial) and analyze the effectiveness of specific treatment (derotation osteotomy) with respect to the extent of asymmetry and overall gait function (e.g. improving lever arm dysfunction) pre- and postoperatively.

## Data Availability

All data generated or analyzed during this study are included in this article. Supplementary data are available on reasonable request.
